# Increases in Genistein in *Medicago sativa* Confer Resistance against the *Pisum* Host Race of *Acyrthosiphon pisum*

**DOI:** 10.3390/insects10040097

**Published:** 2019-04-01

**Authors:** Erliang Yuan, Hongyu Yan, Jing Gao, Huijuan Guo, Feng Ge, Yucheng Sun

**Affiliations:** 1 State Key Laboratory of Integrated Management of Pest Insects and Rodents, Institute of Zoology, Chinese Academy of Sciences, Beijing 100101, China; yuanerliang@ioz.ac.cn (E.Y.); yanhongyu@ioz.ac.cn (H.Y.); gaojing@ioz.ac.cn (J.G.); guohj@ioz.ac.cn (H.G.); gef@ioz.ac.cn (F.G.); 2 College of Life Sciences, University of Chinese Academy of Sciences, Beijing 100049, China

**Keywords:** aphid, genistein, host race, *Medicago sativa*, plant secondary metabolites, transcriptome

## Abstract

Interspecific interaction with host plants have important consequences for the host race formation of herbivorous insects. Plant secondary metabolites, particularly those that are involved in host races specializing on plants, warrant the theory of host specialization. *Acyrthosiphon pisum* comprises various host races that adapt to different Fabaceae plants, which provides an ideal system for determining the behavioral and physiological mechanisms underlying host-adaptive diversification. The current study evaluated the effects of host transfer on population fitness, feeding behavior and the transcriptome-wide gene expression of the two host races of *A. pisum*, one of which was originally from *Medicago sativa* and the other from *Pisum sativum*. The results showed that the *Pisum* host race of *A. pisum* had a lower population abundance and feeding efficiency than the *Medicago* host race in terms of a longer penetration time and shorter duration times of phloem ingestion when fed on *M. sativa*. In contrast, few differences were found in the population abundance and feeding behavior of *A. pisum* between the two host races when fed on *P. sativum*. Meanwhile, of the nine candidate phenolic compounds, only genistein was significantly affected by aphid infestation; higher levels of genistein were detected in *M. sativa* after feeding by the *Pisum* host race, but these levels were reduced relative to uninfested controls after feeding by the *Medicago* host race, which suggested that genistein may be involved in the specialization of the aphid host race on *M. sativa*. Further exogenous application of genistein in artificial diets showed that the increase in genistein reduced the survival rate of the *Pisum* host race but had little effect on that of the *Medicago* host race. The transcriptomic profiles indicated that the transcripts of six genes with functions related to detoxification were up-regulated in the *Pisum* host race relative to the *Medicago* host race of *A. pisum*. These results suggested that the inducible plant phenolics and associated metabolic process in aphids resulted in their differential adaptations to their Fabaceae host.

## 1. Introduction

Host race is considered to be one of several intermediates in the continuum between polymorphisms and the full species [1]. Aphids (Hemiptera: Aphididae) are a group of phytophagous insects that exclusively consume the phloem sap from host plants. There are approximately 4700 species [2], some of which consist of diverging populations, host races, biotypes, or even potential forms of incipient speciation [3,4]. *Acyrthosiphon pisum*, a specialist aphid that feeds on legume plants, has at least 15 different host races [5]. Each is restricted to one or a few species of Fabaceae and is prone to forming host-specialized populations that show differential preference and fitness on specialized host plants [6,7]. For instance, *A. pisum* populations feeding on two important leguminous plants in North America, *Medicago sativa* and *Trifolium pratense*, are highly specialized and reproductively isolated [8,9]. There are strong indications that some host races of *A. pisum* can successfully colonize their native leguminous hosts but fail to establish a colony on the incompatible leguminous host where other host races are natively specialized. The physiological mechanism involved, however, is largely unclear.

It has become clear that the existence of compatible or incompatible interactions between plants and aphids is attributed to both types of organisms [10]. Aphids deliver salivary effectors into the host to manipulate the host defensive signaling pathway [11]. For example, the Armet protein is conducive to the feeding behavior of *A. pisum* on fava beans because of its ability to suppress host defenses [12]. Similarly, in other aphid species, such as *Myzus persicae*, a salivary protein can alleviate defensive compounds in *Arabidopsis thaliana*. Specifically, Mp55, a salivary protein produced by *M. persicae*, can help aphids withstand defensive compounds, such as the 4-methoxyindol-3-ylmethyl glucosinolate, callose deposition and hydrogen peroxide. Furthermore, the transcriptome evidence showed that the gene expression associated with chemosensory and salivary genes was significantly different among the host races of *A. pisum*, especially when comparing the *Medicago* host race with other host races [7]. Given that the different host races of aphids have enormous variations in saliva composition, their specialized responses to host defenses are of paramount importance in host transformation. These effectors could interfere with the defense-signaling pathway [12,13,14] and accordingly alter plant secondary metabolite levels [15]. Few studies have been conducted to identify differences in secondary metabolite product in response to the infestation of different aphid host races. So far, there has been just one study that elaborates the role of Mp55 in *M. persicae*, which could increase aphid reproduction through inhibiting the accumulation of 4-methoxyindol-3-ylmethylglucosinolate and callose of Arabidopsis [13].

There is increasing evidence showing that secondary metabolites produced by plants can support both the antixenosis and antibiosis mechanisms of resistance to aphids. In the case of antixenosis, the presence or higher concentration of these compounds is associated with avoidance or non-preference by the aphid; in the case of antibiosis, these compounds cause direct negative impacts to the fitness and physiology of the aphids [16,17,18]. For example, the indole glucosinolate class in Brassicaceae and the DIMBOA (2,4-dihydroxy-7-methoxy-1,4-benzoxazin-3-one) compounds in Gramineae [19] can be strongly induced by aphids and prevent constant phloem feeding on host plants [20]. Much about the role of the plant secondary metabolites involved in the formation of aphid host races remains unknown. Recent studies have shown that the *Trifolium* and *Medicago* host races of the pea aphid can modulate the salicylic acid (SA) and jasmonic acid (JA) defense signaling pathways on their respective native host plant, while non-native host races cannot [15]. It was, therefore, speculated that the modification of the phytohormone signaling pathway may subsequently alter the accumulation of plant secondary metabolites that confer different effects on the different host races of aphids. It has been shown that the foliar genistein content in *M. truncatula*, which is detrimental for the survival of aphids, was down-regulated by the green morph but up-regulated by the red morph of the *A. pisum*. This may lead to increased abundance, fecundity, growth, and feeding efficiency of green morph, while the pink morph showed decreased fitness [21]. 

In addition to salivary effectors, aphids employ a range of strategies to enhance their host adaptation, including the enhancement of the detoxification enzymes, which can optimize the metabolism of ingested toxic protein or the secondary metabolites of plants [22]. For example, to cope with nicotine, multiple genes coding for detoxification enzymes of *M. persicae*, including cytochrome P450 monooxygenases (P450s) and glutathione S-transferases (GSTs), were up-regulated. These detoxification enzymes rapidly transform the poisonous substances into nontoxic substances [23]. Furthermore, the *CYP6CY3* gene, which detoxifies nicotine, was induced in a tobacco-adapted clone of *M. persicae*. However, it is still unclear which detoxification-related genes are involved in the specificity adaptation of different aphid host races to host plants. 

To determine why some host races of *A. pisum* could not perform well on non-specialized host plants, it is very important to measure the plant secondary metabolites levels to determine the level of plant defense. The different responses in plant secondary metabolites levels influenced by the two host races would favor the hypothesis that the low performance of *A. pisum* on non-specialized host plants is due to their susceptibility to phenolics. The present study aimed (1) to compare the feeding behavior and population abundance of the *Medicago* and *Pisum* host races of *A. pisum* on their specialized and non-specialized legume hosts; (2) to determine whether the levels of defensive phenolic contents, such as phenols and flavonoids, change in response to aphid feeding and have detrimental effects on the performance of the two host races; and (3) to quantify the transcriptome-wide gene expression of the two host races of *A. pisum* when fed on *M. sativa* and to further screen the candidate genes that are involved in the differential host adaptation between the two host races of *A. pisum*. 

## 2. Materials and Methods

### 2.1. Aphids and Host Plants 

Two host races of *A. pisum* were used in this study. The clone *Pisum* host race was collected from *P. sativum* in Yunnan Province, China. The clone *Medicago* host race was collected from *M. sativa* in Ningxia Province, China. The field-collected colonies (*Medicago* host race and *Pisum* host race) were individually established from a single parthenogenetic female. We verified that these two populations are specialized on different host plants by investigating the aphid population abundance and detecting the aphid feeding behavior using the electrical penetration graph (EPG) technique when fed on their own host plant and the alternative plant. The two field-collected colonies (*Medicago* host race and *Pisum* host race) were reared on their own host plant in the laboratory for more than five years. The two host races of *A. pisum* are well adapted to their own host plant. When we transferred the two host races of aphids to the alternative plant (*Medicago* host race to *P. sativum* and *Pisum* host race to *M. sativa*), we found that they cannot perform well on alternative host plants, especially for the *Pisum* host race on *M. sativa*. Two legume plant species, *M. sativa* and *P. sativum*, were grown in 12-cm diameter plastic pots with a standardized soil mixture (75 g/kg organic carbon; 500 mg /kg N; 200 mg/kg P; 300 mg /kg K) in climate chambers (Safe PRX-450C, Ningbo, China) at 22 ± 0.8 °C, 70% ± 5% relative humidity, and a 16 h light/8 h dark photoperiod. *P. sativum* was grown individually in order to get enough plant material for plant secondary metabolites analyses (approximately 4 leaf whorls), while *M. sativa* was grown three plants per pot (approximately 10 leaf whorls). *P. sativum* was used in experiments of aphid abundace 12 days after sowing, and *M. sativa* was used 25 days after sowing [15].

### 2.2. Aphid Population Abundance

To determine how the host plants affected the population abundance of specialized and non-specialized aphid clones, two legume host plants were randomly selected and infested with two apterous 4th instar nymphs. Each aphid–plant combination was replicated six times. The plants were covered with air permeable gauze, and the nymphs developed and produced offspring freely on each plant for 14 days. The aphid numbers were measured 7 and 14 days after infestation. The rearing conditions were the same as those described in Section 2.1.

### 2.3. Aphid Feeding Behavior

The electrical penetration graph (EPG) technique was employed to detect the feeding behaviors of the two host races of *A. pisum* on *M. sativa*. For each EPG recording, a wingless adult aphid was immobilized on ice, and then the aphid dorsum was attached to a gold wire (2 cm in length, 18.5 μm in diameter) using hand-mixed, water-based silver conducting paint glue (EPG Systems). The other side of the gold wire was then glued with a droplet of paint to a copper extension wire (2 cm in length), which was inserted into the input of the EPG head stage amplifier. Another copper electrode (10 cm in length, 2 mm in diameter) was inserted into the soil of the plant container. Aphids were starved for 2 h as an adaptation period between the time of wiring and the beginning of the EPG recording. Aphids were then placed on the abaxial side of the leaf. The plants, aphids and amplifier were placed in a Faraday cage to avoid noise. For each aphid–plant combination, the 8 h EPG waveform recordings of 15 aphids were performed by a Giga-8 DC EPG System (EPG Systems, Wageningen, The Netherlands). The EPG waveforms were manually analyzed using the Stylet + analysis module as previously described: nonpenetration, stylets are outside the plants; pathway, mostly intramural probing activities between mesophyll or parenchyma cells; potential drops (pd), aphids briefly puncture cells during plant penetration; phloem salivation (E1), aphids are injecting watery saliva into the sieve element; phloem ingestion (E2), aphids are ingesting the phloem sap; xylem ingestion (G), aphids are ingesting the xylem sap [24]. 

### 2.4. Plant Material Sampling and Extraction of Phenolics

At the end of the aphid abundance experiment, the aphids were removed from the plants. Above-ground parts of three plant seedlings were harvested and dried. The plants without aphid infestation (called “uninfested”) were used as a control. Each treatment had three replicates. For a typical extraction, approximately 50 mg samples were soaked with 1 mL 70% aqueous MeOH for 1 h in a 60 °C water bath. The extract was centrifuged at 12,000 rpm for 5 min, and the supernatant was filtered by a 0.22-μm filter. The samples were stored in a freezer at −20 °C until chemical analysis. 

### 2.5. Quantification of Plant Secondary Metabolites by HPLC

The foliar phenolic compounds for chemical analysis were determined according to the previous method [25]. We quantified nine phenolic compounds in *M. sativa* by using HPLC: (1) phenolic acids, which includes protocatechuic acid, chlorogenic acid, caffeic acid, 4-hydroxycinnamic acid, syringic acid and ferulic acid; (2) flavonoids, which include rutoside; (3) isoflavones, which includes genistein and genistin. The determination of compounds was performed on a water system with a diode array detector. Chromatograms were registered and integrated at 280, 350, and 254 nm for phenolic acid, flavonoids, and isoflavone, respectively. The mobile phase consisted of 1% H_3_PO_4_–AcN (a linear gradient of 15–100% AcN) with a flow rate of 1 mL/min for 60 min. Compounds were identified by comparing the retention times to those of authentic standards.

### 2.6. Bioassay with Pure Compound 

Artificial diets [26] were used to measure the effects of genistein on the survival of the two host races of *A. pisum*. All chemicals were purchased from Sigma-Aldrich (Missouri, MO, USA). Genistein was added into the diet at 0, 1 and 10 μg/mL concentrations. A diet without genistein, called “0”, was used as a control. There were five biological replicates for each treatment. The solutions were enclosed in parafilm stretched across a tube (2 cm in height and 4 cm in diameter). During this experiment, the artificial diets were changed every two days. Twenty 2nd instar nymphs were exposed to a 100 μL diet containing genistein. For each group, the numbers of surviving aphids were counted after five days.

### 2.7. Transcriptomics Analyses

At the end of aphid abundance experiment, the two host races of *A. pisum* collected from *M. sativa* were used for transcriptomics analyses. Three replicates were employed. 

For each replicate, Trizol (Life) was used to extract the total RNA of 10 adults according to the manufacturer’s instructions. The total RNA was sent to the Beijing Genomics Institute (BGI) Company (Shenzhen, China) for an RNA-seq analysis. RNA was checked for purity and integrity using an Agilent 2100 Bioanalyzer. RNA-seq libraries were prepared following Illumina’s protocols and were sequenced on the Illumina HiSeq 4000 sequencer (Illumina, Inc., San Diego, CA, USA) with a 150-bp paired-end reads. At least 10 million clean reads were obtained for each sample. These reads were mapped to the *A. pisum* genome using Hierarchical Indexing for Spliced Alignment of Transcripts (HISAT) version: v0.1.6-beta (Parameters: --phred64 --sensitive --no-discordant --no-mixed-I 1-X 1000) [27]. The genome sequence and gene annotation data sets were downloaded from AphidBase Official Gene Set v2.1 (http://www.aphidbase.com/). The gene expression levels were measured using RNA-Seq by Expectation-Maximization (RSEM) [28]. Differentially expressed genes were analyzed using the Noiseq package [29] with a fold change ≥ 2 and a divergence probability ≥ 0.8. Blast2GO software was used for gene ontology (GO) annotations [30]. The hypergeometric test was performed with whole transcriptome as the reference set and differentially expressed genes as the test set. GO terms with false discovery rate (FDR) corrected *p* values ≤ 0.01 were considered significantly enriched. Kyoto Encyclopedia of Genes and Genomes (KEGG) analyses were performed to identify significantly enriched pathways represented by differentially expressed genes. The hypergeometric test was performed in the same way to that for GO enrichment analysis. The terms were determined as enriched pathways according to FDR corrected *p* values ≤ 0.01 [31]. 

### 2.8. Quantification of Gene Expression

Trizol (Life) was used to isolate the total RNA from the two host races of *A. pisum*, and 1 μg RNA was used to synthesize the cDNAs, by using FastQuant RT Kit with gDNase (TianGen Biotech, Beijing, China). OligoDTs were used for the RT reaction. Real-time quantitative PCR (RT-qPCR) was used to quantify the expression levels of candidate genes of *A. pisum*. Reactions were performed in a PikoReal Real-time PCR Detection System (Thermo Scientific, Vantaa, Finland) using 10 μL reaction mixture which included 5 μL SuperReal PreMix Plus (TianGen Biotech, Beijing, China), 0.5 μL 10 uM forward, 0.5 μL 10 uM reverse primers, 2 μL 10-fold diluted cDNA template, and 2 μL ddH_2_O. The thermo protocol included preheating at 95 °C for 15 min, followed by 40 cycles of denaturation at 95 °C for 30 s, annealing at 60 °C for 30 s, and elongation at 72 °C for 40 s, and then a final cycle at 95 °C for 30 s, at 55 °C for 30 s and at 95 °C for 30 s. A standard curve was derived from serial dilutions to quantify the copy numbers of target mRNAs. The relative level of each target gene was standardized after comparing the copy numbers of target mRNA with copy numbers of the reference gene ribosomal protein *L27* due to its stability of expression [32]. The specific primers for the genes were designed from *A. pisum* expressed sequence tags using PRIMER6 software (Table S1, Supporting information). There were three biological replicates for each host race. Each biological replicate contained four technical repeats. The fold-changes of the candidate genes were calculated by using the 2^−ΔΔCt^ normalization method. 

### 2.9. Statistical Analysis

Statistical analyses were performed with SPSS 18.0. Student’s *t*-tests were used to analyze the aphid abundance, aphid feeding behavior, and quantification of gene expression for two-group comparisons. A one-way ANOVA was used to analyze the amount of secondary plant metabolites of *M. sativa* and the survival of the two host races of *A. pisum* that were fed on the diet with genistein. The differences were considered to be statistically significant when *p* < 0.05. All of the data were checked for normality and equality of residual error variances and were appropriately transformed if needed to satisfy the assumptions of an analysis of variance.

## 3. Result

### 3.1. Aphid Performance

**Figure 1:**
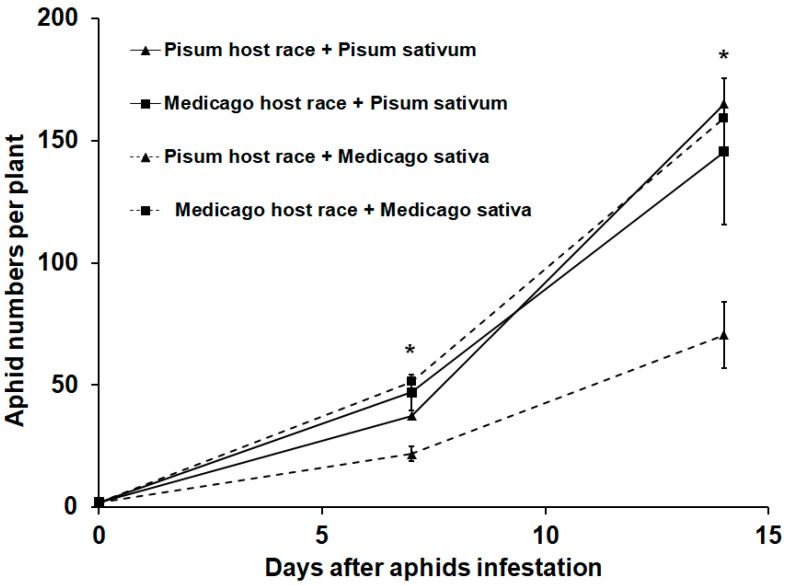
Population abundance of the two host races of *Acyrthosiphon pisum* on *Pisum sativum* and *Medicago sativa*. Each value represents the average (±SE) of the six replicates. “*” indicates significant differences between the *Pisum* host race and *Medicago* host race on *M. sativa*, as determined by Student’s *t*-test at *p* < 0.05.

There was little difference in aphid abundance between the two host races when fed on *P. sativum*. The *Medicago* host race of *A. pisum* exhibited a higher population abundance than the *Pisum* host race at one-week post-infestation (1.4-fold larger than the *Pisum* host race, *p* < 0.05) and two weeks post-infestation (1.3-fold larger than the *Pisum* host race, *p* < 0.05) when fed on *M. sativa* (Figure 1).

The EPG data showed that, before the stylet reached the phloem, the *Pisum* host race of *A. pisum* spent more time in potential drops (aphids briefly puncture cells during plant penetration) than the *Medicago* host race (16 min vs. 27 min), as indicated by the “total pd” in Table 1. The time for the stylet to reach the phloem did not differ between the two host races, as indicated by the “time to first E1”. Regarding the phloem-related parameters, the total duration of the E2 (aphids are ingesting the phloem sap) waveform were significantly reduced on average, from approximately 239 min in the *Medicago* host race to 123 min in the *Pisum* host race, as indicated by the “total E2”. The total duration of E1 (aphids are injecting watery saliva into the sieve element) and the number of E1 or E2 did not differ between the two host races. The remaining parameters had little significance between the two host races of *A. pisum* (Table 1).

### 3.2. Concentrations of Foliar Phenolics in M. sativa

**Figure 2:**
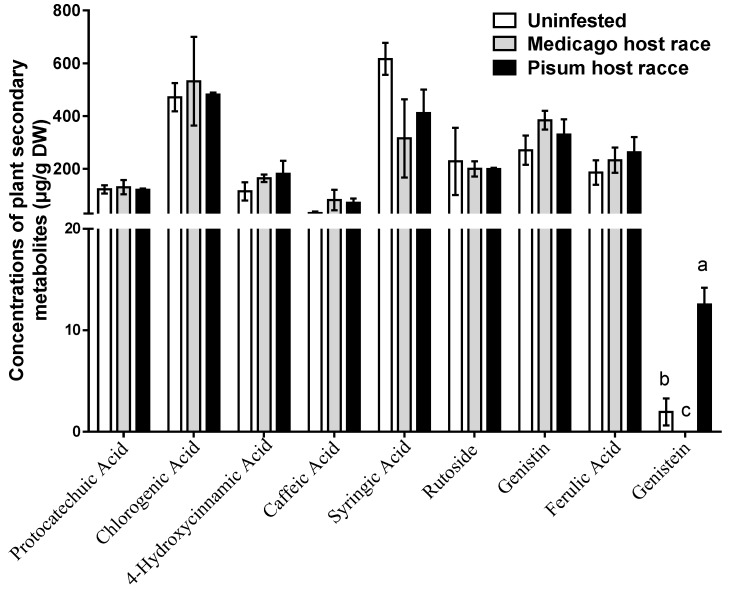
Concentrations of phenolics in the leaves of *Medicago sativa* with the infestation of the two host races of *Acyrthosiphon pisum*. The level of genistein induced by the *Medicago* host race was not detected. Each value represents the average (±SE). Different lowercase letters indicate significant differences among the aphid treatments, as determined by Tukey’s multiple range tests at *p* < 0.05.

To explore the response of plant phenolics to the *Medicago* host race and the *Pisum* host race of *A. pisum* infestation, we analyzed the levels of the phenolics of *M. sativa*. The results showed that the foliar genistein content in *M. sativa* significantly increased when infested by the *Pisum* host race (5.4-fold larger than uninfested treatment, *p* < 0.05) but decreased in response to infestation by the *Medicago* host race relative to the uninfested plants (*p* < 0.05). Furthermore, the content of other foliar phenolics were unaffected by infestation with *A. pisum* (Figure 2).

**Figure 3:**
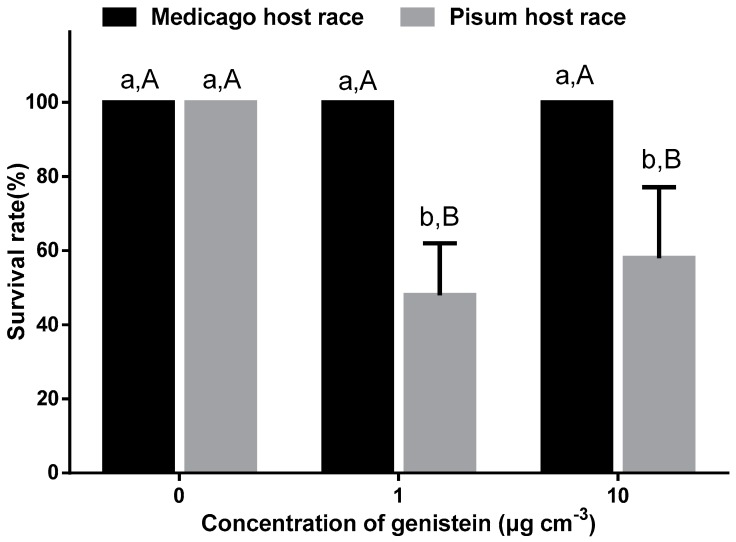
Survival rate of the two host races of *Acyrthosiphon pisum* fed on artificial diets with the application of different concentrations of genistein. Each value represents the mean (±SE) of five replicates. “0” indicates the diets without genistein, which were used as a control. Different uppercase letters indicate significant differences among the genistein treatments within the same host races, as determined by Tukey’s at *p* < 0.05. Different lowercase letters indicate significant differences between the two host races within the same genistein treatment, as determined by Student’s *t*-test at *p* < 0.05.

To determine the effect of genistein on the two host races of *A. pisum* and the different responses of the two host races, we tested their survival rate on the diet with genistein. The exogenous application of 1 μg/cm^−3^ or 10 μg/cm^−3^ genistein into the artificial diet significantly reduced the survival rate of the *Pisum* host race of *A. pisum* relative to the control without genistein (*p* < 0.05), but the two concentrations of genistein did not affect those of the *Medicago* host race. There was no significant difference between the two host races of *A. pisum* when they fed on the diet without genistein. The survival rate of the *Pisum* host race of *A. pisum* was lower than *Medicago* host race with diets containing 1 μg/cm^−3^ (*p* < 0.05) or 10 μg/cm^−3^ (*p* < 0.05) genistein (Figure 3).

### 3.3. Transcriptomic Characteristics of the Two Host Races of A. pisum on M. sativa 

**Figure 4:**
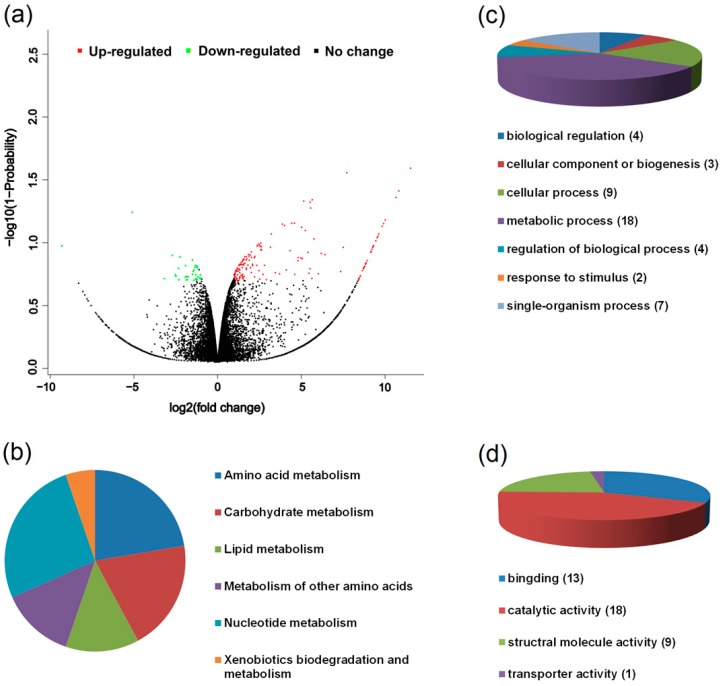
Summary of the sequence annotation of differently expressed genes between the two host races of *Acyrthosiphon pisum* on *Medicago sativa* based on (**a**) a volcano plot of the differently expressed genes, (**b**) KEEG pathway analyses, (**c**) biological control and (**d**) molecular function. “Up-regulated” or “Down-regulated” indicates genes are higher or lower in the *Pisum* host race compared to the *Medicago* host race. “No change” indicates genes with no significant difference between the two host races.

RNA-seq was used to compare the global gene expression profiles between the two host races of *A. pisum* fed on *M. sativa*. After quality check, approximately 44.1, 45.2, and 45.4 million reads were obtained from the three replicates of the *Medicago* host race and 45.1, 44.1, and 44.7 million resds from the *Pisum* host race. The average mapping percentage of raw reads to the reference transcripts was 79% (Table S2, Supporting information). The overall variation in gene expression between the two host races was high. A total of 198 genes were differentially expressed between the two host races of *A. pisum* with a probability > 0.8 (probability, an odds value of 4:1 means that the gene is four times more likely to be differentially expressed than nondifferentially expressed) and a fold change threshold ≥ 2 (Figure 4a). Of the 198 genes in the two host races samples, 161 genes had higher expression in the *Pisum* host race relative to the *Medicago* host race, and 37 genes had lower expression (Tables S3 and S4, Supporting information). There were 67 genes coding for hypothetical proteins with unknown functions. Those (131) that shared homology with genes of known function in the database were categorized according to biological processes and molecular function (Figure 4c,d). Significantly enriched GO categories for genes with higher expression in the *Pisum* host race compared to the *Medicago* host race included biological regulation, cellular component organization or biogenesis, cellular process, metabolic processes, and the regulation of biological process response to stimulus, signaling, single-organism process, binding, catalytic activity, structural molecule activity, and transporter activity (Table S5, Supporting information). For the genes with lower expression in the *Pisum* host race compared to the *Medicago* host race, enriched GO categories included cellular component organization or biogenesis, cellular process, metabolic process, response to stimulus, single-organism process, binding, catalytic activity, and structural molecule activity (Table S6, Supporting information). The KEGG metabolic pathway analyses indicated that the very abundant categories for the differential expression of genes between the two host races on *M. sativa* were amino acid metabolism, carbohydrate metabolism, lipid metabolism, xenobiotics biodegradation and metabolism and nucleotide metabolism (Figure 4b). 

Among the differentially expressed genes between the two host races of *A. pisum* on *M. sativa*, we identified six genes associated with detoxification (Table 2), which included *UDP-glucuronosyltransferase 2B2* (LOC 100166729), *UDP-glucuronosyltransferase 2B17* (LOC 100169601), *UDP-glucuronosyltransferase 1-7C-like* (LOC 100159691), *glutathione-S-transferase* (LOC100570856), *cytochrome P450 6a13* (LOC100572007) and *cytochrome P450 family 6* (LOC100569567). These genes showed higher expression in the *Pisum* host race samples relative to the *Medicago* host race samples with a probability ≥ 0.8 and a fold change ≥ 2.

**Figure 5:**
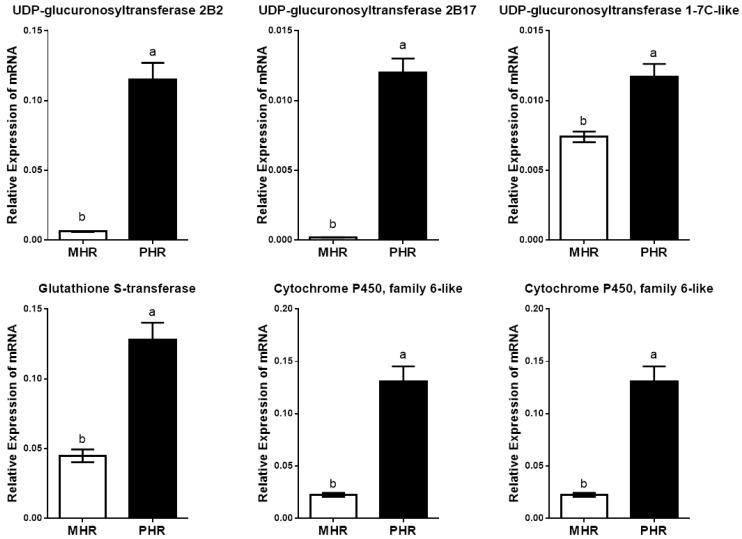
Expression levels of six candidate genes between the two host races of *Acyrthosiphon Pisum on Medicago sativa*. Different lowercase letters indicate significant differences between the *Medicago* host race (MHR) and the *Pisum* host race (PHR), as determined by Student’s *t*-test at *p* < 0.05.

Consistent with the transcriptomic data, further qPCR experiments also confirmed that these six genes were all up-regulated in the *Pisum* host race when compared with the *Medicago* host race (Figure 5).

## 4. Discussion

Divergent natural selection from host plant [33] or facultative symbionts [34] is generally regarded as the driving force of host race formation in insects. Thus, deciphering the unique traits under divergent selection is an essential step towards understanding host race formation [35]. We have shown that, since the *Medicago* host race of *A. pisum* down-regulated while the *Pisum* host race up-regulated the content of genistein, the *Medicago* host race had a higher population abundance and feeding efficiency on *M. sativa* than the *Pisum* host race. Furthermore, the up-regulation of six genes related to detoxification was observed in the *Pisum* host race of *A. pisum*, which suggests a physiological cost in the life history of the *Pisum* host race on *M. sativa*. This study provides a revolutionary insight into the interspecific interactions between the two host races of *A. Pisum* and their Fabaceae hosts. 

The *Medicago* host races of *A. pisum* seem to be capable of colonizing a range of Fabaceae hosts with optimal fitness on their own host *M. sativa*, as well as other hosts, such as *P. sativum*. By contrast, the *Trifolium* host races and *Pisum* host races of *A. pisum* do not establish well on *M. sativa*. Further experiments on phytohormone-dependent resistance suggested that the *Medicago* host races impaired the JA signaling pathway of *M. sativa* by reducing the formation of 12-oxophytodienoic acid (OPDA), while the *Pisum* host races and *Trifolium* host races did not [15]. Moreover, the unique pattern of the aphid host races in manipulating the host defenses may result from the differentially expressed salivary proteins of aphids, which has important consequences for modifying the cross-talk between SA and JA signaling pathways [36]. A recent study showed that Armet, a salivary protein from *A. pisum*, can suppress the effective JA pathway through the modification of the SA pathway to benefit the feeding activity of *A. pisum* [37]. Mp10, a salivary protein from *M. persicae*, could activate both the JA and SA signaling pathways, which led to the relatively low fecundity upon *Nicotiana benthamiana* [36]. It was suggested that the failure of suppression in efficient host defenses may result in a lower population fitness of the *Pisum* host race on *M. sativa*. Additionally, there are strong indications that some facultative endosymbionts of *A. pisum* contribute to host plant colonization. For example, most of the *Trifolium* host race of the *A. pisum* carried *Regiella insecticola* [38,39,40,41,42]. Once *R. insecticola* was removed, the fitness of the *Trifolium* host race of *A. pisum* was remarkably decreased when fed on *T. repens*. Nevertheless, inconsistent results also revealed that the acquisition of *R. insecticola* had little effect on improving the performance of some lineages of the *Trifolium* host race of *A. pisum* on *T. pratense* [43,44,45].

Plant phenolics include flavonoid glycosides, chlorogenic acid, and caffeic acid, which act as antifeedants or exhibit toxicity activity against aphid feeding, which leads to oxidative stress in the aphid tissues and attenuates aphid digestion and metabolism [46,47,48,49]. A previous study found that the application of genistein in an artificial diet prolonged the period of probing and shortened the passive ingestion duration of *A. pisum*, which suggested that genistein decreased the feeding efficiency of *A. pisum* [50]. Similar detrimental effects of genistein were also shown in other aphid species, i.e., *Aphis glycines* and *Aphis craccivora* [51,52]. Our result showed that the infestation of the *Pisum* host race induced the accumulation of genistein in *M. sativa*, which, in turn, reduced the survival rate of aphids and suggested that genistein is one of the components that are responsible for decreased fitness on *M. sativa*. Furthermore, the higher content of foliar genistein may result from longer penetration and probing durations of the *Pisum* host race, which causes stronger induced defenses in *M. sativa*. The antibiosis effects of rutoside and ferulic acid against phloem-sucking insects have been reported in soybean and barley [53,54]. Although these compounds were detected in our study, our data showed that there was little difference in the concentration of rutoside and ferulic acid between the two host races, suggesting these phenolics are likely not associated with resistance to the *Pisum* host race in *M. sativa*. 

The strategies of phenolic metabolism in aphids include the avoidance absorption by the gut, elimination from the body cavity, and degradation by detoxifying enzymes [18]. A previous study showed that the genistein content in *A. pisum* would increase with the rise of genistein content in *M. truncatula*, which lead to the low performance of *A. pisum* on *M. truncatula* [21]. It is indicated that decreasing the accumulation of phytotoxins in the body cavity is an efficient way for aphids to adapt to plant secondary metabolites. Our study found that the *Pisum* host races had shorter phloem ingestion duration on *M. sativa* than those of the *Medicago* host races, which suggested that reduced feeding in *M. sativa* could effectively function to reduce the consumption and absorption of genistein by the *Pisum* host race. 

Recent transcriptome data showed that a subset of chemosensory and salivary proteins may have an important role in the host-race formation of *A. pisum* [7]. Our transcriptome data found that the detoxification genes of aphids may participate in their host transfer and colonization. The GSTs and P450s of aphids are involved in the detoxification of toxic host plant allelochemicals [55,56,57]. For example, aphid GSTs have been reported to detoxify glucosinolates in Arabidopsis, nicotine in tobacco and the hydroxamic acid in cereal plants [23,58,59]. The detoxification-related genes were highly up-regulated in the *Pisum* host race, suggesting that a higher expression of detoxification enzymes is required for the *Pisum* host race to cope with *M. sativa*. This may cause a physiological cost, which reduces the fitness of the *Pisum* host race on *M. sativa*. A more detailed understanding of the molecular mechanisms of genistein degradation in aphids are needed to refine the utilization of the gene silencing approach for the future control of aphid infestation. 

## Figures and Tables

**Table 1:** The feeding behavior of the two host races of *Acyrthosiphon pisum* when fed on *Medicago sativa*.

	*Medicago sativa*	
Parameters	*Medicago* Host Race	*Pisum* Host Race
Nonpenetration ^1^	47.00 ± 10.20 a	86.29 ± 10.20 a
Pathway ^2^	148.75 ± 25.86 a	175.79 ± 25.86 a
Total pd (potential drops) ^3^	14.12 ± 2.02 **b**	26.66 ± 2.02 **a**
Time to first pd	8.60 ± 3.79 a	8.45 ± 4.83 a
Number of pds before first E1	65.09 ± 10.08 a	96.43 ± 10.08 a
Total E1 (phloem salivation) ^4^	32.26 ± 7.98 a	21.29 ± 7.98 a
Number of E1	4.85 ± 0.96 a	4.57 ± 0.96 a
Time to first E1	100.64 ± 17.50 a	125.90 ± 17.50 a
Total E2 (phloem ingestion) ^5^	231.70 ± 35.11 **a**	123.25 ± 35.11 **b**
Number of E2	2.73 ± 0.51 a	1.79a ± 0.35 a
Time to first E2	168.96 ± 30.98 **b**	248.06 ± 30.98 **a**
Total G (xylem ingestion) ^6^	6.16 ± 3.43 a	21.37 ± 8.85 a

^1^ Nonpenetration, stylets are outside the plants; ^2^ Pathway, mostly intramural probing activities between mesophyll or parenchyma cells; ^3^ potential drops(pd), aphids briefly puncture cells during plant penetration; ^4^ phloem salivation (E1), aphids are injecting watery saliva into the sieve element, ^5^ phloem ingestion (E2), aphids are ingesting the phloem sap. ^6^ xylem ingestion (G), aphids are ingesting the xylem sap. Values are the mean (±SE) of 12 biological replicates. Different lowercase letters indicate significant differences between the *Pisum* host race and *Medicago* host race (Non-parametric Mann–Whitney test; *p* < 0.05).

**Table 2:** Selected related genes differentially expressed between the *Medicago* host race and *Pisum* host race.

Gene ID	Putative Function	log2 Fold Change ^1^	Probability ^2^
LOC100166729	*UDP-glucuronosyltransferase 2B2*	4.42 × up	0.93
LOC100169601	*UDP-glucuronosyltransferase 2B17*	6.40 × up	0.88
LOC100159691	*UDP-glucuronosyltransferase 1-7C-like*	2.58 × up	0.89
LOC100570856	*glutathione S-transferase*	1.74 × up	0.87
LOC100572007	*cytochrome P450, family 6-like*	2.61 × up	0.89
LOC100569567	*cytochrome P450, family 6*	2.47 × up	0.83

^1^ Log2 Fold change compared to the *Medicago* host race. ^2^ Probability, an odds value of 4:1 means that the gene is four times more likely to be differentially expressed than non-differentially expressed.

## Supplementary Materials

The following are available online at https://www.mdpi.com/2075-4450/10/4/97/s1. Table S1: Primer sequences for quantitative PCR; Table S2: Summary of transcriptome parameters of *Pisum* host race samples and *Medicago* host race samples; Table S3: Genes with higher expression in the *Pisum* host race samples when compared to the *Medicago* host race samples; Table S4: Genes with lower expression in the *Pisum* host race samples when compared to the *Medicago* host race samples; Table S5: Significantly enriched Gene Ontology (GO) categories for genes with higher expression in the *Pisum* host race samples relative to the *Medicago* host race samples (FDP corrected *p* < 0.01); Table S6: Significantly enriched Gene Ontology (GO) categories for genes with lower expression in the *Pisum* host race samples relative to the *Medicago* host race samples (FDP corrected *p* < 0.01).
